# Retraction: Exosomes derived from microRNA-148b-3p-overexpressing human umbilical cord mesenchymal stem cells restrain breast cancer progression

**DOI:** 10.3389/fonc.2024.1422567

**Published:** 2024-05-14

**Authors:** Frontiers Editorial Office

The Publisher retracts the cited article.

“Following publication, concerns were raised regarding the validity of the data in the article. The authors were unresponsive to our requests for raw data during the investigation, which was conducted in accordance with Frontiers’ policies. Given the concerns, and the lack of raw data, the editors no longer have confidence in the findings presented, and this article is retracted.

This retraction was approved by the Specialty Chief Editor of Frontiers in Oncology - Cancer Molecular Targets and Therapeutics and the Chief Executive Editor of Frontiers. The authors received a communication regarding the retraction and had a chance to respond. This communication is recorded by the publisher.”

